# Role of Gut Microbiota-Generated Short-Chain Fatty Acids in Metabolic and Cardiovascular Health

**DOI:** 10.1007/s13668-018-0248-8

**Published:** 2018-09-28

**Authors:** Edward S. Chambers, Tom Preston, Gary Frost, Douglas J. Morrison

**Affiliations:** 1 Section for Nutrition Research, Faculty of Medicine, Imperial College London, 6th Floor, Commonwealth Building, Hammersmith Hospital, London, UK; 2 Stable Isotope Biochemistry Laboratory, Scottish Universities Environmental Research Centre, University of Glasgow, East Kilbride, Glasgow, G75 0QF Scotland

**Keywords:** Short-chain fatty acids, Gut microbiome, Fermentation, Glucose homeostasis, Blood pressure, Appetite regulation, Obesity, Inflammation, Metabolic health, Cardiovascular disease

## Abstract

**Purpose of this Review:**

This review assesses the latest evidence linking short-chain fatty acids (SCFA) with host metabolic health and cardiovascular disease (CVD) risk and presents the latest evidence on possible biological mechanisms.

**Recent Findings:**

SCFA have a range of effects locally in the gut and at both splanchnic and peripheral tissues which together appear to induce improved metabolic regulation and have direct and indirect effects on markers of CVD risk.

**Summary:**

SCFA produced primarily from the microbial fermentation of dietary fibre appear to be key mediators of the beneficial effects elicited by the gut microbiome. Not only does dietary fibre fermentation regulate microbial activity in the gut, SCFA also directly modulate host health through a range of tissue-specific mechanisms related to gut barrier function, glucose homeostasis, immunomodulation, appetite regulation and obesity. With the increasing burden of obesity worldwide, the role for gut microbiota-generated SCFA in protecting against the effects of energy dense diets offers an intriguing new avenue for regulating metabolic health and CVD risk.

## Introduction

Cardiovascular disease (CVD) is the leading cause of death worldwide, accounting for one in three deaths in the USA [1]. Although significant declines in CVD mortality have been observed in developed countries in the late twentieth century [2, 3] owing largely to improvements in public health and healthcare, the burden of CVD still remains high in low and middle-income countries where over three out of four CVD deaths worldwide occur [4]. Diet and lifestyle-related risk factors associated with CVD include smoking, physical inactivity, obesity, diabetes mellitus, dyslipidemia and hypertension. Recent declines in CVD mortality are however in danger of being reversed with the increased prevalence of obesity. Since the 1980s, the world has seen the prevalence of obesity double and recent worldwide data suggests that there are now more people overweight than underweight globally and in all regions except parts of sub-Saharan Africa and Asia [5]. Looking ahead, in the UK alone, it is forecast that by 2050, 60% of males and 50% of females will be obese [6]. The current and continuing burden of obesity in developed countries and the emerging burden in low and middle-income countries pose a significant challenge to continued decline in CVD mortality.

An emerging area of interest in metabolic health and its link with CVD risk is the gut microbiome. The advent of high-throughput metagenomic techniques has facilitated new insights into the role of the gut microbiome in CVD risk [7]. Importantly, mechanistic insights into the causal pathways are now emerging for microbially mediated production of metabolites that can have both beneficial and detrimental effects on metabolic health and CVD risk [8]. For example, microbially mediated trimethylamine production and subsequent hepatic modification to trimethylamine N-oxide (TMAO) has emerged as a strong microbiome-mediated risk factor for CVD [9–11]. Recent faecal microbial transplant work has demonstrated that TMAO production can by modified by targeting the gut microbiome [12•] highlighting the potential of the gut microbiome as a modifiable therapeutic target. The possibilities for modulating gut microbiome activity through dietary, biological or xenobiotic approaches makes this “organ” an attractive target for a range of host health outcomes, with potential for cost-effective individual and population scale interventions. The primary function of the gut microbiome is to process undigested material eluting from the small intestine which includes undigested dietary components and material secreted into the intestine (that remains undigested) by the host including pancreatic secretions, bile acids, mucins and material sloughed from the small intestine through the normal passage of intestinal contents. Of significant biological interest in metabolic health is the role of dietary fibre and the primary products of their breakdown by the gut microbiota, the short-chain fatty acids (SCFA).

### Formation and Primary Function of SCFA in Human Health

**Fig. 1:**
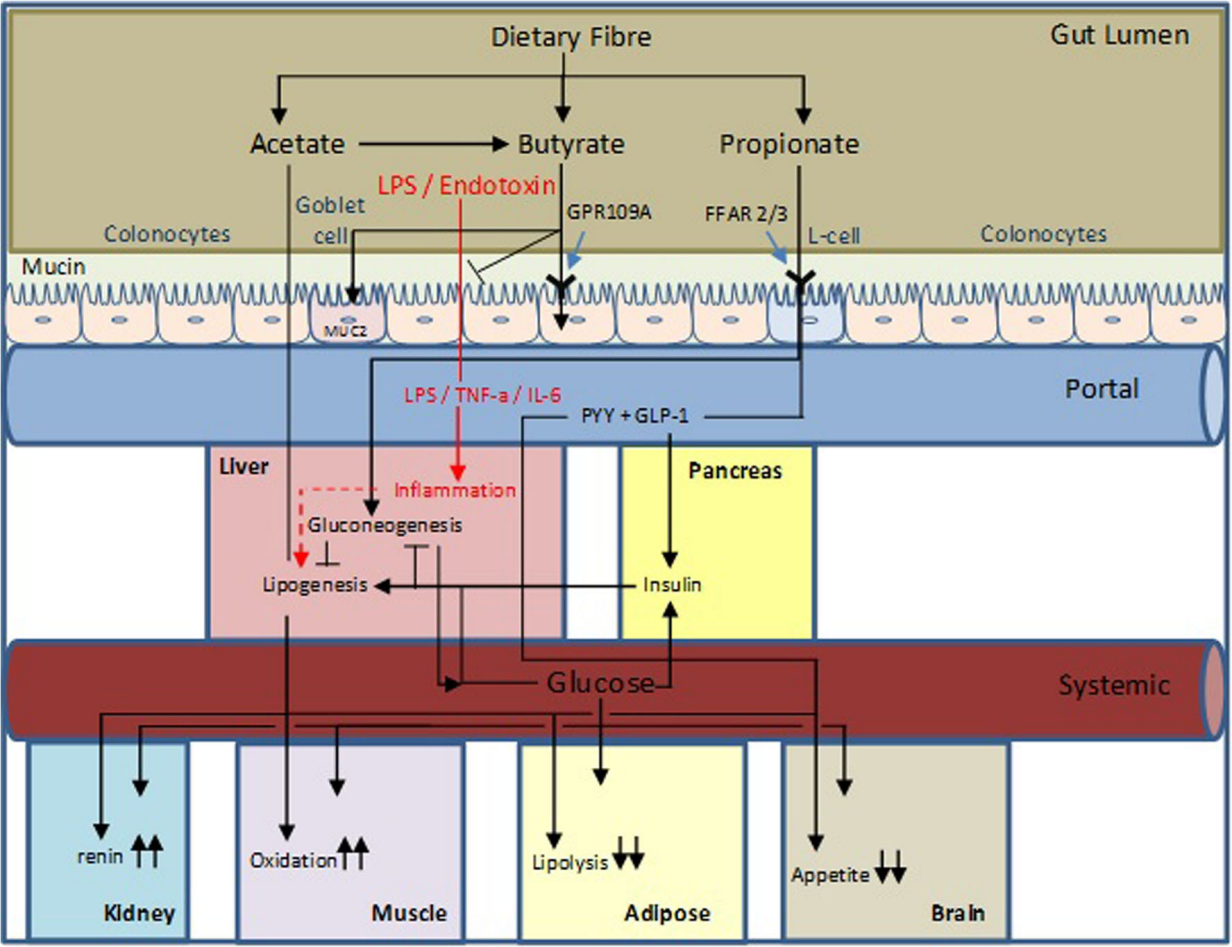
Overview of the mechanisms of action of SCFA in metabolic health and CVD. Acetate produced in microbial fermentation in the gut largely escapes first-pass metabolism in the liver. It can contribute acetyl units to lipogenesis in the cytosol of hepatocytes and adipocytes but its primary site of oxidation is peripheral muscle. It can also regulate adipose tissue lipolysis and can act on central appetite regulation. Propionate acts locally in the gut on enteroendocrine L-cells to stimulate release of the anorexigenic gut hormones PYY and GLP-1. Propionate is largely absorbed across the intestine and sequestrated primarily in the liver where it can be oxidised or used in gluconeogenesis. Butyrate is largely oxidised at the gut epithelium where it plays a central role in orchestrating the tight junction protein complexes to control gut barrier function. It also plays role in regulating inflammatory cell populations and function through receptor-mediated and histone deacetylation mechanisms. All three SCFA potentially play a role in blood pressure regulation; acetate and propionate through a complex interplay involving renin production mediated through Olfr78 and counter-regulation through FFAR3 and butyrate through attenuation of angiotensin II-induced expression of renal prorenin receptors and renin

A number of recent epidemiological studies have highlighted the inverse association between dietary fibre intake and CVD risk factors [13–18]. Non-digestible carbohydrates (NDC) are an important fraction of dietary fibre and SCFA are the main products of saccharolytic fermentation of NDC in the large intestine. Acetate, propionate and butyrate are the primary SCFA products and are produced in the approximate molar ratio of 60:20:20 reaching a combined concentration of over 100 mM in the intestinal lumen, although circulating concentrations are much lower for propionate and butyrate especially [19]. The balance between saccharolytic fermentation and proteolytic fermentation is primarily related to dietary intake and NDC availability to the microbiota. In elegant work, diet switching experiments have demonstrated rapid changes in microbial metabolic activity and diversity related to the protein, lipid and NDC (dietary fibre) content of the diet [20••, 21]. Thus, the microbiome has become an attractive target because of the ease of its modulation by diet, with the aim of altering host response. SCFA play an important role in host health beyond the recovery of energy from undigested food. Butyrate plays an important role in orchestrating the integrity of the large bowel and small intestinal barrier and supplying energy to epithelial cells in the large intestine [22]. Recent work has also demonstrated a role for butyrate in regulating immune response through expansion of Treg cell populations [23, 24] adding to a body of earlier work on the role of SCFA in ameliorating the pro-inflammatory response of immune cells to antigen stimulus (reviewed in [25]). Propionate largely passes across the gut lumen, although a recent study suggests a role for propionate in intestinal gluconeogenesis [26], where it is almost quantitatively sequestrated in the liver where it may act as a gluconeogenic substrate or be oxidised [27]. Some acetate is converted to butyrate by lumenal bacteria; however, acetate largely escapes splanchnic extraction and is available to peripheral tissues where it can be used for lipogenesis in adipose tissue or oxidised by muscle [28]. The role of SCFA, including the tissue-specific metabolism of SCFA, has recently been reviewed elsewhere [29]. The complex luminal, splanchnic and peripheral cell-specific nature of SCFA action and sequestration is illustrated in Fig. 1.

### Role of SCFA in Metabolic and Cardiovascular Health

SCFA have a number of potential roles in modulating metabolic health and CVD risk factors through direct and indirect routes.

#### Blood Pressure Regulation

Possibly the most direct route of modulating CVD risk is SCFA modulation of systolic blood pressure (SBP) and diastolic blood pressure (DBP). A recent controlled trial in humans has demonstrated a potential adjuvant effect of butyrate in the reduction of DPB through a reduction in inflammation [30], and the abundance of butyrate-producing bacteria has been inversely associated with blood pressure and with plasminogen activator inhibitor-1 levels in early pregnancy [31]. Indirect evidence from dietary fibre studies, that should be treated with caution as to the causal role of SCFA because of the pleiotropic effects of dietary fibre, demonstrate that dietary fibre is associated with reduction in BP in Type 1 diabetes (albeit at higher levels of fibre consumption) [32]. A recent meta-analysis concluded that all dietary fibre types when considered together reduced BP (− 0.9 and − 0.7 mmHg for SBP and DBP, respectively) although the effect size was greater for beta-glucan (− 2.9 and − 1.5 mmHg for SDP and DBP, respectively)-type fibres [33]. The mechanism by which dietary fibres elicit these effects remain to be fully elucidated.

#### Metabolic Regulation

An area where the role of SCFA is gaining much interest is their effects in obesity and metabolic regulation (glucose and lipid homeostasis). Epidemiological and robust animal data demonstrates an inverse relationship between dietary fibre intake and adiposity and weight gain. Recent studies are beginning to provide insight into the role of SCFA. Acetate plays a role in central appetite regulation [34], and increasing production in the distal colon may be more effective than the proximal colon, promoting fat oxidation, improved glucose homeostasis and inflammatory status [35•]. The role of acetate in energy homeostasis and substrate metabolism has recently been reviewed elsewhere [36]. Our recent work using an inulin propionate ester to target deliver to the large intestine has demonstrated in controlled human trials that propionate directly attenuates appetite and food intake [37], influences food choice [38], improves pancreatic function [39] and modulates hepatic lipid accretion [40]. Oral propionate has also been shown to increase fat oxidation in humans [41]. Acute oral, but not intravenous, butyrate has been shown to reduce food intake and improve glucose and lipid profiles via a gut-brain neural circuit in animals [42]. In overweight/obese men, colonic infusions of SCFA appear to increase fat oxidation, energy expenditure and PYY release, and decrease adipose tissue lipolysis [43]. Inulin, a fructo-oligosaccharide prebiotic NDC which is readily fermented producing SCFA, has demonstrated beneficial effects on adiposity [44] substrate metabolism [45], insulin sensitivity [46] and appetite regulation [47–49] although the effects seen in humans are inconsistent and may be related to the levels of inulin intake [50]. A recent meta-analysis tentatively concluded that inulin-type fructans elicit a beneficial effect on lipid profiles and glucose metabolism [51].

#### Gut Barrier Function

Perhaps the most intriguing role for SCFA that may have the greatest impact on host metabolic health is their importance in orchestrating the epithelial barrier to maintain gut integrity and prevent the translocation of bacterial pro-inflammatory molecules across the gut wall. Of the SCFA produced in the colon, current evidence points towards a key regulatory role for butyrate. A plethora of animal studies have promoted a key role for butyrate in maintaining epithelial integrity [52, 53] and restoring normal barrier function in challenge models of disease [54–57] primarily through orchestration of the tight junction proteins which govern paracellular permeability and solute transport through the channels between intestinal cells [58]. The consequence of maintaining an optimal gut barrier is preventing the translocation of microbial cell wall components, like lipopolysaccharide, which are strongly pro-inflammatory. An additional pivotal role for butyrate is the induction of mucin production which creates a physical barrier between luminal bacterial and epithelial cells [59]. Interestingly, in a recent human study examining gut permeability in hypertension, markers of increased gut permeability and LPS were elevated in hypertensive individuals and a strong correlation was observed between gut permeability and SBP [60•]. The study was only able to demonstrate a protective role for butyrate however in an associated mouse model experiment. Inulin, which is rapidly fermented to SCFA, improves intestinal function in mice [61] but improvements in barrier function were not observed in humans [62] which create uncertainty about translation of findings from animals to humans.

#### Gut Microbial Function

Recent evidence from animal studies suggest that soluble dietary fibre reduces TMA and TMAO metabolism by 40.6 and 62.6%, respectively, which was associated with increased SCFA production and reduced serum lipids and cholesterol [63]. In contrast, inulin supplementation in humans had no effect on fasting or post-prandial TMAO levels [64], indicating the challenges in translating findings from animals to humans and the complexity of the role of different dietary fibres on microbial activity.

### Mechanistic Insights into The Role of SCFA in Metabolic and Cardiovascular Health

#### SCFA Are Ligands for Several Receptors

A number of now de-orphaned G protein coupled receptors have been identified for which SCFA act as natural ligands [65, 66]. Free fatty acids receptors (FFAR) 2 and 3, which have higher affinity for acetate and propionate, are expressed in the intestine, adipose tissue, pancreas and a number of immune cell subtypes. GPR109A has higher affinity for butyrate and may play a role in inflammatory pathways in the intestine [67]. Olfactory receptor 78 (Olfr78) receptors have higher affinity for acetate and propionate and are localised in autonomic nerves in the heart and gut, in the smooth muscle cells of arteries and in renal juxtaglomerular cells [68•]. SCFA also function as histone deacetylase inhibitors (HDACs). Histone acetylation is a key regulator of transcription factor activation and downstream gene expression through regulation of chromatin structure.

#### Blood Pressure

The role of SCFA in the regulation of blood pressure has been examined, mainly in animal models. Acetate and propionate appear to regulate blood pressure in a complex interplay involving induction of renin production through Olfr78 and counter-regulation through FFAR3. Evidence from knock out models suggests that propionate induces release of renal renin and a rise in BP through Olfr78 but can also induce a reduction in BP that appears FFAR3 dependent [68•, 69]. In rats, butyrate has been shown to lower BP by attenuating angiotensin II-induced expression of renal prorenin receptors and renin [70]. However, given the low circulating concentration of butyrate in humans [71], whether this pathway is physiologically relevant requires further research to elucidate. Circulating concentrations of propionate and butyrate in humans are generally < 10 μmol/L [71] whereas in animal studies concentrations examined vary from physiological < 10 μmol/L [70] to supra-physiological > 0.1–10 mmol/L concentrations [68•].

#### Gut Barrier Function

SCFA and butyrate in particular have long been recognised as important substrates for maintaining a healthy gut. Butyrate is the preferred substrate for colonocytes. More recently, a role for SCFA in regulating epithelial integrity through co-ordinated regulation of tight junction proteins which regulate the intracellular molecular highway between the lumen and hepatic portal system has been postulated. Hyperglycaemia and increased gut permeability are associated with translocation of bacteria and/or their cell wall components which trigger an inflammatory cascade that has been associated with obesity and insulin resistance [72•]. In mice, butyrate can also act on nucleotide-binding oligomerization domain-like receptors (NLRs), key modulators of inflammation in an FFAR2 dependent manner to regulate key components of the tight junction complex [73]. Work in cell models has revealed a possible role for p38 MAPK [74], IL-10 receptor mediated [75] and AMPK/intracellular ATP [76] regulation of claudin proteins by butyrate. Intriguingly, the involvement of FFAR2 in this pathway opens up a role for other SCFA in regulating barrier function also [77]. In a high-fat diet-induced steatohepatitis mouse model, butyrate was observed to attenuate steatohepatitis through improvement in high-fat diet-induced intestinal mucosa damage, upregulation of zonulin and reduced endotoxin levels [78]. These improvements were associated with downregulation of endotoxin-associated genes (TLR4 and Myd88) and expression of pro-inflammatory genes (MCP-1, TNF-α, IL-1, IL-2, IL-6 and IFN-γ) in the liver. Given the emerging critical role of maintaining a competent physical barrier in the gut between the luminal bacteria and the host immune system, well-designed human studies are warranted to elucidate the role of SCFA in gut barrier function.

#### Appetite Regulation and Energy Intake

SCFA have been suggested to protect against diet-induced obesity by reducing appetite and energy intake. However, a number of studies have reported that incorporating SCFA into the diet of rodents has no effect on food intake [26, 79]. Similarly, in humans, directly incorporating propionate into the diet had no effect on energy intake at an ad libitum test meal or over the 24-h period following consumption [80]. Oral SCFA are rapidly absorbed from the upper gastrointestinal tract, thus may not markedly raise concentrations in the gut lumen. This would appear to be important for SCFA to promote an effect on appetite regulation.

A recent study by Li et al. reported a reduction in energy intake following intragastric administration of butyrate but not through intravenous administration of butyrate [42]. This disparity in results may be due to the fact that intragastric administration allows butyrate to reach its natural site of production in the gut lumen and therefore interact with intestinal receptors, which is not achieved with peripheral administration. Using a targeted approach to delivering propionate to the large intestine, we have demonstrated that propionate induces appetite regulation, reduced food intake and prevent weight gain in humans [37].

The available rodent and human studies therefore suggest that oral SCFA supplementation does not modulate appetite responses, whilst delivering SCFA to more distally in the gut may reduce energy intake. The SCFA receptors FFAR2 and FFAR3 are co-expressed in glucagon-like peptide 1 (GLP-1) and peptide YY (PYY) expressing cells [65], leading to the suggestion that SCFAs might reduce energy intake via stimulating the release of these anorectic hormones. Several studies using in vitro models of enteroendocrine cell lines have investigated this effect of SCFAs on gut hormone release [81]. These reports highlight that SCFAs can stimulate anorectic gut hormone release via FFAR2. It has also been suggested that high levels of SCFA in the lower gut could modulate energy intake via gut-brain neural circuits. For example, De Vadder et al. reported that elevated colonic propionate production could induce vagal signalling in the gut or portal vein via FFAR3 [26]. Similarly, Li et al. found that the decrease in food intake following intragastric administration of butyrate in mice was blocked after vagotomy [42].

In summary, studies that have targeted delivery of SCFA to the GI tract have shown reductions in energy intake, which may be related to the anorectic gut hormone release and/or direct neural gut-brain signalling via FFAR2 and FFAR3 receptors. SCFA may also modulate body weight and obesity by increasing energy expenditure. Indeed, a number of studies have reported that both acute and chronic administration of SCFAs promotes energy expenditure in rodents [42, 82, 83]. Available studies in humans have also shown that colonic [43] and oral SCFA [41] supplementation raises rates of energy expenditure. It has consistently been reported that the increase in energy expenditure stimulated by SCFA is associated with a promotion in whole-body lipid oxidation [41, 43, 79]. The increase in energy expenditure and lipid oxidation by SCFA administration has been postulated to be due to stimulation of sympathetic nervous system (SNS) activity, via FFAR3 expressed at the level of the sympathetic ganglion [84], an increase in brown adipose tissue (BAT) activity [42] and via suppression of PPARγ in peripheral tissues, which upregulates lipid oxidation [79].

#### Glucose Homeostasis

SCFA have important effects on glucose homeostasis through a range of mechanisms. As previously described, improved gut barrier function reduces inflammation and oxidative stress promoting improved insulin sensitivity (reviewed in [85]). In dietary fibre supplementation studies in humans, increased plasma propionate has been associated with a reduction in post-prandial insulin [86] and improved glucose homeostasis [37] through improved pancreatic β-cell function [39] although supplementation with resistant starch observed improvements in glucose homeostasis that appeared to be independent of circulating SCFA and were explained by decreased free fatty acid output [87]. Acetate and butyrate may also play a role in maintaining β-cell function through their action on cytotoxic T cells, mediated via B-cells and direct action of SCFA on regulatory T cell populations [88•].

#### Obesity

SCFA protect against diet-induced obesity through a number of mechanisms. SCFA produced in the colon stimulate FFAR 2/3 on enteroendocrine L-cells in the colon leading to release of the anorexigenic gut hormones GLP-1 and PYY [81, 89]. The role of acetate in appetite regulation, adiposity and weight gain is controversial. Frost et al. demonstrated that acetate induced central appetite regulation and reduced food intake in mice protecting against diet-induced weight gain [34]. More recently, Perry et al. have demonstrated that acetate has the opposite effect, leading to increased glucose-stimulated insulin secretion, increased ghrelin secretion, hyperphagia and obesity in mice [90]. These conflicting results require further work to decipher but may be associated with metabolic status and site of acetate administration [36].

Using a targeted approach to delivering propionate to the large intestine, we have demonstrated that propionate induces appetite regulation, reduced food intake and prevent weight gain in humans that may in part be explained by increased GLP-1 and PYY [37]. In mice, SCFA have been shown to decrease PPARγ expression and activity leading to increased expression of mitochondrial uncoupling protein 2 and increased AMP-to-ATP ratio, stimulating oxidative metabolism in the liver and adipose tissue via AMPK [79]. Butyrate may also play a role in preventing diet-induced obesity through increasing energy expenditure by activating of β3 adrenergic receptor mediated lipolysis in white adipose tissue [82] and through activation of the adiponectin-mediated pathway and stimulation of mitochondrial function in the skeletal muscle [91]. In a type 2 diabetes mouse model, butyrate—a potent HDAC inhibitor—attenuated myocyte apoptosis, reduced production of reactive oxygen species and increased angiogenesis through MKK3/p38/PRAK activation [92]. Accumulation of fat in the liver is associated with impaired hepatic insulin sensitivity and is associated with type 2 diabetes [93]. In humans [40] and mice [94], propionate has been associated with preventing hepatic lipid accumulation through suppression of genes involved in fatty acid synthesis and potentially through competition for intracellular co-enzyme A stores.

## Conclusions and Future Perspectives

There is quite compelling evidence from animal models that SCFA can play an important role in regulating metabolic health and mitigating CVD risk. However, careful interpretation of the evidence is necessary particularly when using global genetic knockout models because of the tissue-specific nature of expression of receptors for SCFA. Furthermore, cognisance must be made of the physiologically relevant concentrations that various tissues are exposed to. Whilst oral supplementation or gavage with SCFA is practical and attractive for animal studies, this is generally not how SCFA appear in the gut in humans. The site and rate of SCFA production in the gut may be critical to the physiological consequences and therefore modelling human physiology is important to correctly interpreting animal studies. Better still, well-controlled human intervention studies are needed to develop a strong evidence base if the research and clinical community are to be convinced of the beneficial role SCFA play in human health. With emerging effects around the importance of SCFA for the regulation of barrier function and inflammation, it is critical that we understand the relevance of these effects for disease mitigation because there are relatively inexpensive options to intervene at the population level. The role of SCFA in managing adiposity and body weight gain is well documented in animals and the emergent role of SCFA in appetite regulation and obesity in humans also presents an exciting opportunity to intervene at a population level to tackle perhaps the most pressing health issue of our time—obesity.
